# Supplementary material for: Discovery of the 1-naphthylamine biodegradation pathway reveals a broad-substrate-spectrum enzyme catalyzing 1-naphthylamine glutamylation
Source: eLife. 2024 Aug 20;13:e95555. doi: 10.7554/eLife.95555 (PMC11335346; doi:10.7554/eLife.95555)
Supplement: Figure 4—figure supplement 1—source data 2. [file elife-95555-fig4-figsupp1-data2.zip › Figure_4-figure_supplement_1-source_data_2/Figure_4-figure_supplement_1-source_data_2.docx]

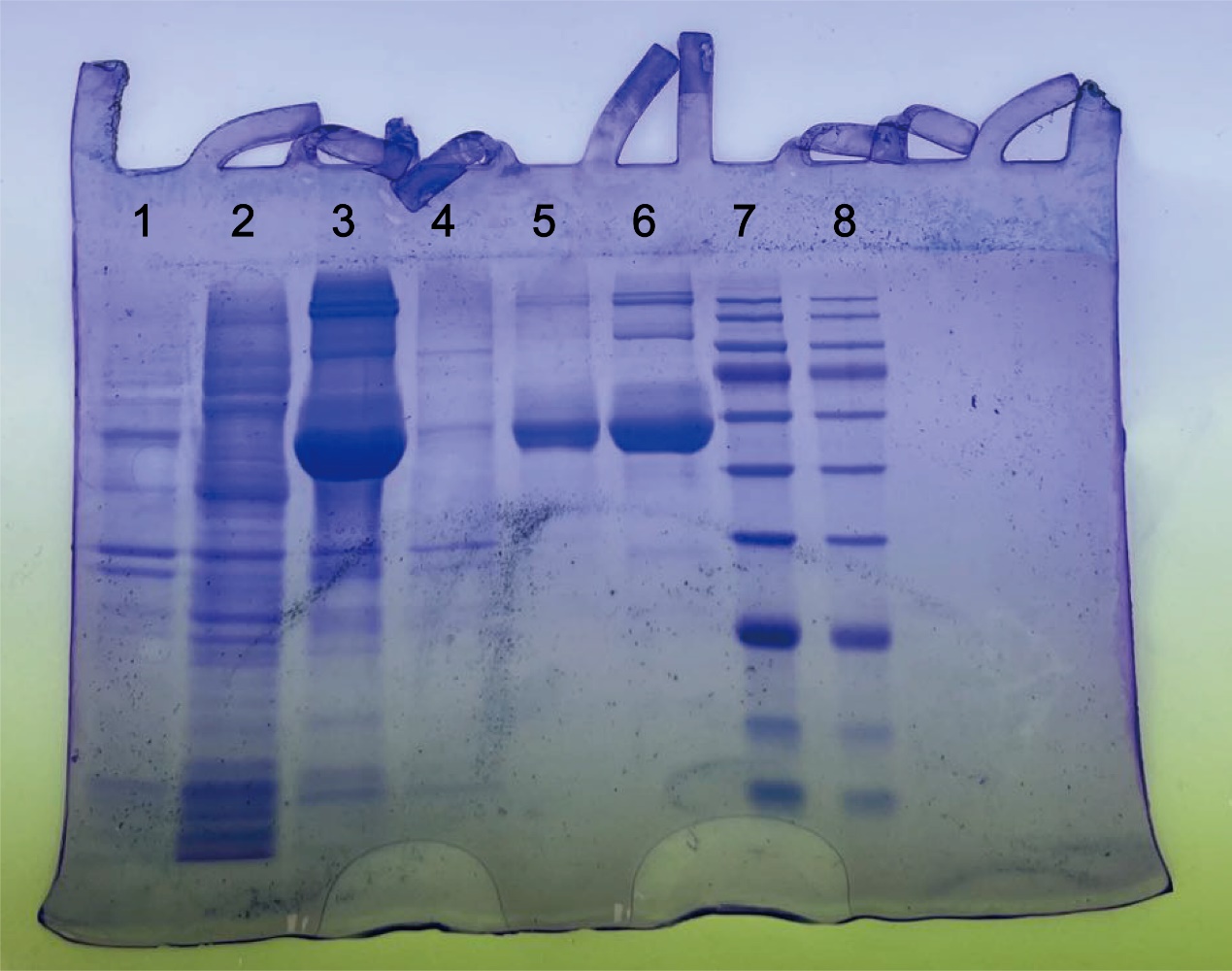


Figure 4-figure supplement 1-source data 2: Uncropped image of the gel shown in Figure 4-figure supplementary 1B. Lane 1: whole-cell extract; lane 2: pellet after cell lysis; lane 3: crude extract of cells; lane 4: flow-through from StrepII affinity column; lane 5: peak “b” after gel filtration chromatography (as shown in Figure_4-figure_supplement_1); lane 6: peak “a” after gel filtration chromatography (as shown in Figure_4-figure_supplement_1); lanes 7-8: molecular weight marker.
