# Supplementary figures and images for: Discovery of the 1-naphthylamine biodegradation pathway reveals a broad-substrate-spectrum enzyme catalyzing 1-naphthylamine glutamylation
Source: eLife. 2024 Aug 20;13:e95555. doi: 10.7554/eLife.95555 (PMC11335346; doi:10.7554/eLife.95555)

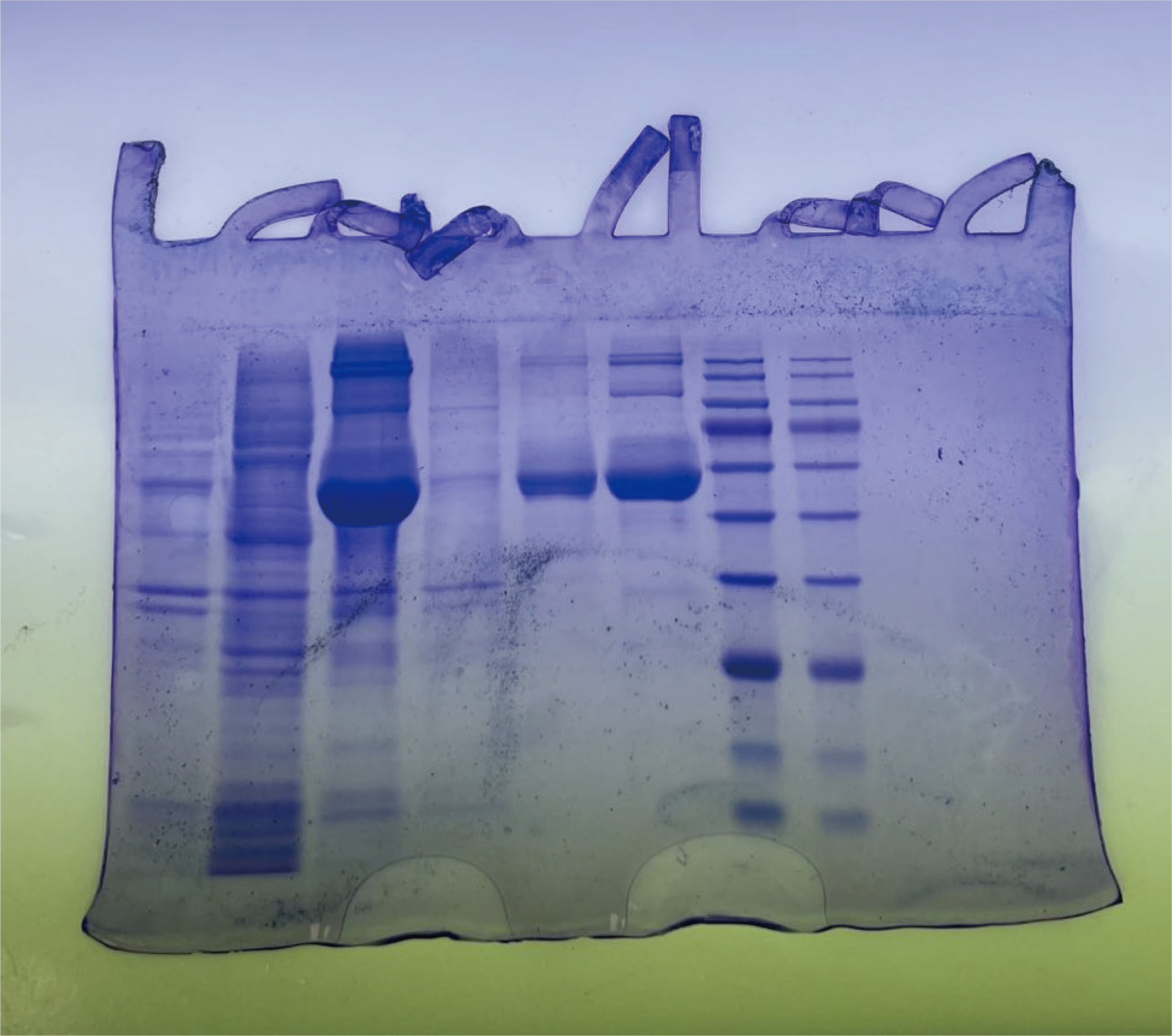

Supplement: Figure 4—figure supplement 1—source data 3. [file elife-95555-fig4-figsupp1-data3.zip › Figure_4-figure_supplement_1-source_data_3.tif]
