# Supplementary material for: Discovery of the 1-naphthylamine biodegradation pathway reveals a broad-substrate-spectrum enzyme catalyzing 1-naphthylamine glutamylation
Source: eLife. 2024 Aug 20;13:e95555. doi: 10.7554/eLife.95555 (PMC11335346; doi:10.7554/eLife.95555)
Supplement: Supplementary file 1. [file elife-95555-supp1.docx]

Supplementary File 1. Identities of ORFs from gene clusters responsible for 1NA degradation

| Gene | Homologous protein; source | Accession no. | Identity (%) | Proposed function of gene product |
| --- | --- | --- | --- | --- |
| *npaA1* | AtdA1; *Acinetobacter* sp. YAA | BAA13010.1 | 91 | γ-glutamylnaphthylamide synthase |
| *npaA2* | AtdA2; *Acinetobacter* sp. YAA | BAA13011.1 | 98 | γ-glutamylnaphthylamide amidotransferase enzyme |
| *npaA3* | alpha subunit of oxygenase component AtdA3; *Acinetobacter* sp. YAA | BAA13012.1 | 98 | alpha subunit of oxygenase component of γ-glutamylnaphthylamide dioxygenase |
| *npaA4* | beta subunit of oxygenase component AtdA4; *Acinetobacter* sp. YAA | BAA13013.1 | 90 | beta subunit of oxygenase component |
| *npaA5* | ferredoxin reductase AtdA5; *Acinetobacter* sp. YAA | BAA13014.1 | 72 | ferredoxin reductase |
| *npaB* | *cis*-naphthalene dihydrodiol dehydrogenase NagB;  *Ralstonia* sp. U2 | AAD12612.1 | 47 | truncated *cis*-naphthalene dihydrodiol dehydrogenase |
| *npaF* | aldehyde dehydrogenase NagF; *Ralstonia* sp. U2 | AAD12613.1 | 97 | aldehyde dehydrogenase |
| *npaC* | 1,2-dihydroxynaphthalene dioxygenase NagC; *Ralstonia* sp. U2 | AAD12614.1 | 97 | 1,2-dihydroxynaphthalene dioxygenase |
| *npaX* | transposase of type 3 insertion sequence family;  *Burkholderia multivorans* | WP_105776820.1 | 67 | transposase of type 3 insertion sequence family |
| *npaE* | *trans*-*O*-hydroxybenzylidenepyruvate hydratase-aldolase NagE; *Ralstonia* sp. U2 | AAD12616.1 | 96 | *trans*-*O*-hydroxybenzylidenepyruvate hydratase-aldolase |
| *npaD* | 2-hydroxychromene-2-carboxylate isomerase NagD;  *Ralstonia* sp. U2 | AAD12617.1 | 100 | 2-hydroxychromene-2-carboxylate isomerase |
| *npaJ* | glutathione-S-transferase-like protein NagJ; *Ralstonia* sp. U2 | AAD12618.1 | 99 | glutathione-S-transferase-like protein |
| *npaI* | gentisate 1,2-dioxygenase NagI; *Ralstonia* sp. U2 | AAD12619.1 | 100 | gentisate 1,2-dioxygenase |
| *npaK* | fumarylpyruvate hydrolase NagK; *Ralstonia* sp. U2 | AAD12620.1 | 100 | fumarylpyruvate hydrolase |
| *npaL* | maleylpyruvate isomerase NagL; *Ralstonia* sp. U2 | AAD12621.1 | 100 | maleylpyruvate isomerase |
| *npaG* | salicylate 1-hydroxylase NahG; *Pseudomonas putida* G7 | BAE92165.1 | 77 | salicylate 1-hydroxylase |
| *npaR* | transcriptional activator NahR; *Pseudomonas putida* G7 | P10183.2 | 79 | transcriptional activator |
| *catI* | 3-oxoadipate CoA-transferase subunit A CatI;  *Pseudomonas* *knackmussii* B13 | Q8VPF3.1 | 94 | 3-oxoadipate CoA-transferase subunit A |
| *catJ* | 3-oxoadipate CoA-transferase subunit B CatJ;  *Pseudomonas knackmussii* B13 | Q8VPF2.1 | 96 | 3-oxoadipate CoA-transferase subunit B |
| *catF* | beta-ketoadipyl-CoA thiolase CatF;  *Pseudomonas knackmussii* B13 | Q8VPF1.1 | 90 | beta-ketoadipyl-CoA thiolase |
| *catD* | 3-oxoadipate enol-lactonase 2 CatD;  *Acinetobacter baylyi* ADP1 | P00632.3 | 48 | 3-oxoadipate enol-lactonase 2 |
| *catA* | catechol 1,2-dioxygenase CatA;  *Acinetobacter baylyi* ADP1 | P07773.1 | 53 | catechol 1,2-dioxygenase |
| *catC* | muconolactone delta-isomerase CatC;  *Pseudomonas aeruginosa* PAO1 | Q9I0X4.1 | 84 | muconolactone delta-isomerase |
| *catB* | *cis,cis*-muconate lactonizing enzyme I CatB;  *Pseudomonas putida* | P08310.4 | 85 | *cis,cis*-muconate lactonizing enzyme I |
| *catR* | transcriptional regulator CatR;  *Pseudomonas putida* | P20667.1 | 69 | transcriptional regulator |
