# Supplementary material for: Discovery of the 1-naphthylamine biodegradation pathway reveals a broad-substrate-spectrum enzyme catalyzing 1-naphthylamine glutamylation
Source: eLife. 2024 Aug 20;13:e95555. doi: 10.7554/eLife.95555 (PMC11335346; doi:10.7554/eLife.95555)
Supplement: Supplementary file 2. [file elife-95555-supp2.docx]

Supplementary File 2. Crystallographic data collection and refinement of NpaA1 and its complex.

|  | **Apo—NpaA1** | **NpaA1—AMP PNP** | **NpaA1—ADP—MetSox-P** |
| --- | --- | --- | --- |
| **Pdb code** | 8X6Z | 8WWU | 8WWV |
| **Data collection** |  |  |  |
| Space group | P 21 21 21 | P 21 21 21 | P 21 21 21 |
| Cell dimensions |  |  |  |
| a, b, c (Å) | 121.64 140.14 218.77 | 123.68 140.57 217.03 | 122.40 140.54 216.44 |
| α, β, γ (°) | 90 90 90 | 90 90 90 | 90 90 90 |
| Resolution (Å) | 19.98-2.95 (3.01-2.95) | 49.69-2.00 (2.03-2.00) | 36.86-2.30 (2.34-2.30) |
| *R*_sym_ | 0.228 (1.034) | 0.11 (0.52) | 0.12 (0.95) |
| *R*_pim_ | 0.097 (0.433) | 0.06 (0.31) | 0.05 (0.39) |
| *I/σI* | 9.4 (2.0) | 10.6 (2.7) | 15.7 (3.4) |
| Completeness (%) | 99.6 (100.0) | 98.6 (91.9) | 99.9 (99.9) |
| Redundancy | 7.1 (7.4) | 7.3 (6.2) | 13.3 (13.6) |
| CC(1/2) | 0.989 (0.762) | 0.996 (0.891) | 0.999 (0.936) |
| **Refinement** |  |  |  |
| Resolution (Å) | 19.98-2.95 | 43.82-2.00 | 36.86-2.30 |
| No. reflections | 78923 | 250611 | 321667 |
| *R*_work_/*R*_free_ (%) | 22.38/24.69 | 18.05/20.70 | 21.19/23.17 |
| No. atoms |  |  |  |
| Protein | 22241 | 22991 | 22951 |
| Ligand/ion | 10 | 282 | 282 |
| Water | 63 | 2109 | 657 |
| *B*-factors |  |  |  |
| Protein | 46.33 | 29.49 | 49.35 |
| Ligand/ion | 82.97 | 32.23 | 33.58 |
| Water | 32.33 | 36.63 | 43.33 |
| **R.m.s. deviations** |  |  |  |
| Bond lengths (Å) | 0.007 | 0.01 | 0.01 |
| Bond angles (°) | 1.09 | 1.36 | 1.32 |
| **Ramachandran analyses** | | | |
| Favored (%) | 95.62 | 98.94 | 99.08 |
| Allowed (%) | 4.17 | 1.06 | 0.51 |
| Disallowed (%) | 0.21 | 0.00 | 0.00 |
