# Supplementary material for: Discovery of the 1-naphthylamine biodegradation pathway reveals a broad-substrate-spectrum enzyme catalyzing 1-naphthylamine glutamylation
Source: eLife. 2024 Aug 20;13:e95555. doi: 10.7554/eLife.95555 (PMC11335346; doi:10.7554/eLife.95555)
Supplement: Supplementary file 3. [file elife-95555-supp3.docx]

Supplementary File 3. Strains and plasmids used in this study

| Strain or plasmid | Characteristics | Reference or source |
| --- | --- | --- |
| Strains |  |  |
| *Escherichia coli* DH5α | *supE44lacU169*(Ф80*lacZ*ΔM15) *recA1* *endA1* *hsdR17 thi-1* | Novagen |
| *E*. *coli BL21*(DE3) | F^-^ *ompT* *hsdS* (r_B_^-^ m_B_^-^) *gal* *dcm* *lacY1*(DE3) | Novagen |
| *Pseudomonas* sp. JS3066 | 1-naphthylamine utilizer | This study |
| *P. putida* KT2440-Δ*cat*AΔ*ggt* | KT2440 mutant; catechol and γ-glutamylanilide degradation negative |  |
| Plasmids |  |  |
| pBBR1MCS-2 | Km^r^; broad-host-range expression vector | (Kovach et al., 1995) |
| pNPA01 | 5.1-kb fragment containing *npaA1* to *npaA5* in pBBR1MCS-2 | This study |
| pNPA01-Δ*A1* | pNPA01 derivative lacking *npaA1* | This study |
| pNPA01-Δ*A2* | pNPA01 derivative lacking *npaA2* | This study |
| pNPA01-Δ*A12* | pNPA01 derivative lacking *npaA1A2* | This study |
| pET-29a(+) | Km^r^; expression vector | Novagen |
| pET-*npaA1* | Km^r^; the *npaA1* gene cloned into pET-29a(+) | This study |
| pET-*npaG* | Km^r^; the *npaG* gene cloned into pET-29a(+) | This study |
| pET-atd*A1* | Km^r^; the *atdA1* gene cloned into pET-29a(+) | (Ji et al., 2019) |

**References**

Kovach, M. E., Elzer, P. H., Hill, D. S., Robertson, G. T., Farris, M. A., Roop, R. M., 2nd, & Peterson, K. M. (1995). Four new derivatives of the broad-host-range cloning vector pBBR1MCS, carrying different antibiotic-resistance cassettes. *Gene*, *166*(1), 175-176. <https://doi.org/10.1016/0378-1119(95)00584-1>

Ji, J., Zhang, J., Liu, Y., Zhang, Y., Liu, Y., & Yan, X. (2019). The substrate specificity of aniline dioxygenase is mainly determined by two of its components: glutamine synthetase-like enzyme and oxygenase. *Appl Microbiol Biotechnol*, *103*(15), 6333-6344. <https://doi.org/10.1007/s00253-019-09871-3>
