# Supplementary material for: Discovery of the 1-naphthylamine biodegradation pathway reveals a broad-substrate-spectrum enzyme catalyzing 1-naphthylamine glutamylation
Source: eLife. 2024 Aug 20;13:e95555. doi: 10.7554/eLife.95555 (PMC11335346; doi:10.7554/eLife.95555)
Supplement: Supplementary file 4. [file elife-95555-supp4.docx]

Supplementary File 4. Primers used in this study.

| Primer | Sequence (5’→3’) | Purpose |
| --- | --- | --- |
| NpaF | GTCGACGGTATCGATAAGCTTATGAGCCGAAAATTTGATTTTATTAC | Amplification of *npaA1A2A3A4A5* for expression |
| NpaR | CGCTCTAGAACTAGTGGATCCTTATTGGTCGAAGGATATTTCTATATTTT |  |
| NpaF-ΔA1 | GTCGACGGTATCGATAAGCTTATGTCTAAACGCTTTGCATTATTGT | Amplification of *npaA2A3A4A5* for expression |
| NpaR-ΔA1 | CGCTCTAGAACTAGTGGATCCTTATTGGTCGAAGGATATTTCTATATTTT |  |
| NpaF1-ΔA2 | GTCGACGGTATCGATAAGCTTATGAGCCGAAAATTTGATTTTATTAC | Amplification of *npaA1A3A4A5* for expression |
| NpaR1-ΔA2 | GGTTTTCATATTGACCTCTGAAAATCACAGTAAGTTG |  |
| NpaF2-ΔA2 | CAGAGGTCAATATGAAAACCATAAATCAACTAATTCAGTC |  |
| NpaR2-ΔA2 | CGCTCTAGAACTAGTGGATCCTTATTGGTCGAAGGATATTTCTATATTTT |  |
| NpaF-ΔA12 | GTCGACGGTATCGATAAGCTTATGAAAACCATAAATCAACTAATTCAGTC | Amplification of *npaA3A4A5* for expression |
| NpaR-ΔA12 | CGCTCTAGAACTAGTGGATCCTTATTGGTCGAAGGATATTTCTATATTTT |  |
| NpaA1-F1 | TAAGAAGGAGATATACATATGTGGAGCCACCCGCAGTTCGA | Amplification of *npaA1* for expression |
| NpaA1-F2 | CAGTTCGAAAAGGATGACGACGACAAGATGAGCCGAAAATTTGATTTTATTAC |  |
| NpaA1-R | CTCGAGTGCGGCCGCAAGCTTTCACAGTAAGTTGAAGTATTCTTTTTGTTC |  |
| NpaG-F1 | CAGTTCGAAAAGGATGACGACGACAAGATGGGTACGAACATGAGCAAGAA | Amplification of *npaG* for expression |
| NpaG-F2 | TAAGAAGGAGATATACATATGTGGAGCCACCCGCAGTTCGA |  |
| NpaG-R | CTCGAGTGCGGCCGCAAGCTTTCAGGCGCAGCGGCTGA |  |
