# Supplementary material for: Discovery of the 1-naphthylamine biodegradation pathway reveals a broad-substrate-spectrum enzyme catalyzing 1-naphthylamine glutamylation
Source: eLife. 2024 Aug 20;13:e95555. doi: 10.7554/eLife.95555 (PMC11335346; doi:10.7554/eLife.95555)
Supplement: Supplementary file 5. [file elife-95555-supp5.docx]

Supplementary File 5. Primers used for constructing mutant vectors.

| Primers | Oligonucleotide sequences (5’ to 3’) |
| --- | --- |
| V201A-F | CAAGGGTACTCCGCACTACTT |
| V201A-R | ATGTTCAAGTAGTGCGGAGTA |
| V201C-F | CAAGGGTACTCCTGTCTACTT |
| V201C-R | ATGTTCAAGTAGACAGGAGTA |
| V201D-F | CAAGGGTACTCCGATCTACTT |
| V201D-R | ATGTTCAAGTAGATCGGAGTA |
| V201E-F | CAAGGGTACTCCGAACTACTT |
| V201E-R | ATGTTCAAGTAGTTCGGAGTA |
| V201F-F | CAAGGGTACTCCTTTCTACTT |
| V201F-R | ATGTTCAAGTAGAAAGGAGTA |
| V201G-F | CAAGGGTACTCCGGTCTACTT |
| V201G-R | ATGTTCAAGTAGACCGGAGTA |
| V201H-F | CAAGGGTACTCCCATCTACTT |
| V201H-R | ATGTTCAAGTAGATGGGAGTA |
| V201I-F | CAAGGGTACTCCATTCTACTT |
| V201I-R | ATGTTCAAGTAGAATGGAGTA |
| V201K-F | CAAGGGTACTCCAAACTACTT |
| V201K-R | ATGTTCAAGTAGTTTGGAGTA |
| V201L-F | CAAGGGTACTCCCTGCTACTT |
| V201L-R | ATGTTCAAGTAGCAGGGAGTA |
| V201M-F | CAAGGGTACTCCATGCTACTT |
| V201M-R | ATGTTCAAGTAGCATGGAGTA |
| V201N-F | CAAGGGTACTCCAATCTACTT |
| V201N-R | ATGTTCAAGTAGATTGGAGTA |
| V201P-F | CAAGGGTACTCCCCGCTACTT |
| V201P-R | ATGTTCAAGTAGCGGGGAGTA |
| V201Q-F | CAAGGGTACTCCCAGCTACTT |
| V201Q-R | ATGTTCAAGTAGCTGGGAGTA |
| V201R-F | CAAGGGTACTCCCGTCTACTT |
| V201R-R | ATGTTCAAGTAGACGGGAGTA |
| V201S-F | CAAGGGTACTCCAGCCTACTT |
| V201S-R | ATGTTCAAGTAGGCTGGAGTA |
| V201T-F | CAAGGGTACTCCACCCTACTT |
| V201T-R | ATGTTCAAGTAGGGTGGAGTA |
| V201W-F | CAAGGGTACTCCTGGCTACTT |
| V201W-R | ATGTTCAAGTAGCCAGGAGTA |
